# Draft Genome Sequence of *Enterobacter ludwigii* NCR3, a Heavy Metal–Resistant Rhizobacterium

**DOI:** 10.1128/genomeA.01076-16

**Published:** 2016-10-06

**Authors:** Eleonora Egidi, Jennifer L. Wood, Sanja Aracic, Ruban Kannan, Lachlan McDonald, Carolyn A. Bell, Edward M. Fox, Wuxing Liu, Ashley E. Franks

**Affiliations:** a Department of Physiology, Anatomy and Microbiology, La Trobe University, Bundoora, Victoria, Australia; b CSIRO Agriculture and Food, Werribee, Victoria, Australia; c Key Laboratory of Soil Environment and Pollution Remediation, Institute of Soil Science, Chinese Academy of Sciences, Nanjing, China

## Abstract

We report here the draft genome of *Enterobacter ludwigii* NCR3, a Gram-negative bacterium isolated from the *Carpobrotus rossii* (Haw.) Schwantes rhizosphere. The analysis of the ~4.8-Mb draft genome shows that this strain harbors several genes associated with heavy metal resistance and plant growth–promoting activity, suggesting its potential application in microbe-assisted phytoremediation.

## GENOME ANNOUNCEMENT

A promising approach to optimize the synergistic effect of plants and microorganisms during the phytoremediation of heavy metal (HM)–contaminated sites consists of coupling phytoextraction with soil bioaugmentation (1). The addition of microorganisms can change the bioavailability of metals and simultaneously promote plant growth results in an enhanced metal uptake by plants (2). Therefore, being able to identify microorganisms with the potential to assist the plant-mediated HM environmental remediation is fundamental to improving the process efficiency.

Due to their ability to increase HM mobilization in soils, stimulate plant growth, and influence HM accumulation in plant tissues, species belonging to the family *Enterobacteriaceae* have been previously proposed as suitable candidates to support bioaugmentation-assisted HM remediation (3). Here, we report the draft genome of *Enterobacter ludwigii* NCR3, a plant growth–promoting bacterium isolated from the rhizosphere of the heavy metal hyperaccumulator *Carpobrotus rossii* (Haw.) Schwantes and grown in a cadmium-contaminated environment (4). When tested for HM resistance, NCR3 showed a MIC of 50, 150, and 800 mg·L^−1^ for cadmium, copper and zinc, respectively (4), suggesting tolerance for a wide range of heavy metals.

Genomic DNA was isolated using a DNeasy blood and tissue kit (Qiagen) and converted to sequencing libraries using the Nextera XT DNA library preparation kit (Illumina). Libraries were normalized and pooled before sequencing on an Illumina MiSeq with 2 × 300 paired-end reads. For each isolate, the A5-miseq pipeline (5) was used to perform read trimming and correction, contig assembly, crude scaffolding, misassembly correction, and final scaffolding. No putative misassemblies were detected with this method. Genome sequencing resulted in high-coverage assemblies of the 4,779,466-bp genome (115-fold median coverage; 54.7% G+C content), which should represent most of the functional annotated genes and allow for comparative studies. The final number of contigs was 23 with an *N*_50_ value of 771,694 bp.

The RAST annotation system (6) showed that the *E. ludwigii* NCR3 genome contains 4,498 coding sequences. Consistent with Liu et al. (4), the genome harbors several predicted genes linked to HM resistance. These include the *ars* (*arsRDABC*) operon, as well as genes encoding for the proteins CopC and CopD associated with the copper resistance operon *copABCD*, and the cobalt-zinc-cadmium resistance protein CzcA. Several antibiotic resistance operons were also found (e.g., *mdtABCD*). Interestingly, we identified genes related to organic acids biosynthesis, suggesting the ability of *E. ludwigii* NCR3 to change the metal’s bioavailability and contribute to plant-mediated metal uptake (7). The presence of genes encoding for siderophores (enterobactin and aerobactin) and auxin biosynthesis also confirms that NCR3 possesses characteristics relevant for a beneficial plant–microbe interaction.

### Accession number(s).

This whole-genome shotgun project has been deposited at DDBJ/ENA/GenBank under the accession number MCGF00000000. The version described in this paper is the first version, MCGF01000000.
